# Monolayer TiS_2_ Nanosheets on Au(111)–Structural Characterization and Effect of Edge Stability for Shape Control

**DOI:** 10.1002/smll.202506023

**Published:** 2025-08-03

**Authors:** Niko Kruse, Kerry Hazeldine, Martin Hedevang, Celina Groothuis, Duy Le, Talat S. Rahman, Jeppe V. Lauritsen, Lars Mohrhusen

**Affiliations:** ^1^ Institute of Chemistry Carl von Ossietzky Universität Oldenburg Carl‐von‐Ossietzky Straße 9‐11 D‐26129 Oldenburg Germany; ^2^ Interdisciplinary Nanoscience Center (iNANO) Aarhus University Gustav Wieds Vej 14 Aarhus DK‐8000 Denmark; ^3^ Department of Physics University of Central Florida 4111 Libra Drive Orlando FL 32816‐2385 USA; ^4^ Present address: IMEC Kapeldreef 75 Leuven 3001 Belgium

**Keywords:** 2D nanomaterials, edge stability, scanning tunneling microscopy, transition metal dichalcogenides, X‐ray photoelectron spectroscopy

## Abstract

Transition metal dichalcogenides are promising alternatives to noble metal catalysts, e.g., for (photo‐)activation of greenhouse gases or hydrogenations. Herein, a direct synthetic route for 2D TiS_2_ nanosheets on Au(111) by titanium deposition in the presence of a mild, organic, non‐oxidizing sulfur source is presented. High‐resolution scanning tunneling microscopy (STM) is used to gain atomic‐level insights into the TiS_2_ nanosheet morphology. In contrast to the literature, this protocol gains mostly hexagonal and truncated triangular nanosheets with an increased edge contrast in STM, analog to metallic edge states in MoS_2_. Synchrotron‐based photoelectron spectroscopy allows insights into compositional details, specifically to distinguish different S sites on the TiS_2_ sheets and other S species on the sample. Further, a minimum size is identified (9 S atoms side length), which underlines the importance of moiré reconstructions for stress relief. The TiS_2_ sheets coexist with [Au]Ti_1_S_3_ clusters, in which a single gold atom is alloyed into the surface and capped by three S atoms. Together with the finding of a critical sheet size, this points toward on‐surface Ostwald ripening as a relevant process in the sheet formation. Ab‐initio calculations (density functional theory) underscore that the chemical potential of S is an essential descriptor to maintain shape control.

## Graphical abstract

Scanning tunneling microscopy (STM) and X‐ray photoelectron spectroscopy (XPS) have been used to obtain atomic‐level insights into 2D TiS_2_ nanosheets and [Au]Ti_1_S_3_ nuclei synthesized on Au(111) surfaces. Density functional theory (DFT) calculations showed that the chemical potential of S is the relevant shape descriptor and allows experimental steering between triangular sheets and hexagonal/truncated triangular shapes. This work guides future protocols for the fabrication of tailored 2D nanosulfides.

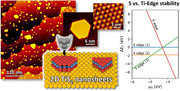

## 1 Introduction

Transition metal dichalcogenides (TMDC) have been intensively studied over the last decades due to their fascinating properties within various applications, such as their optoelectronic characteristics^[^ ^1^ ^]^ or their use as catalysts in the chemical industry.^[^ ^2^, ^3^, ^4^ ^]^ These materials consist of sandwich‐like MX_2_ structures, in which two layers of chalcogens (X = S, Se, Te) are covalently bound to an intralayer of transition metals (M = Mo, Ti, Co, W, etc.).^[^ ^3^ ^]^ Weak van der Waals interactions bond these sandwich layers together, which allows for the exfoliation of 2D layers from bulk materials.^[^ ^5^ ^]^ Several synthetic routes in various environments, from liquids to solid phase, have been reported, including chemical vapor transport processes or top‐down approaches such as sonification or exfoliation from bulk materials.^[^ ^6^, ^7^, ^8^, ^9^, ^10^, ^11^, ^12^, ^13^ ^]^ To understand the material properties on the atomic level, nanostructured MS_2_ model systems on the Au(111) surfaces have been established, for example for CoS_2,_^[^ ^5^ ^]^ RuS_2,_^[^ ^14^ ^]^ WS_2_^[^ ^15^ ^]^ or MoS_2_^[^ ^2^ ^]^ (and the promoted versions CoMoS^[^ ^16^ ^]^ and NiMoS).^[^ ^17^ ^]^ Amongst those, MoS_2_ is by far the best studied, not only because of its unique electronic properties^[^ ^18^ ^]^ but also in view of its commercial application as a hydrotreatment catalyst, e.g., to remove heteroatomic compounds from crude oils (hydrodesulfurization (HDS)) or bio oils (hydrodeoxygenation (HDO), hydrodenitrogenation (HDN)).^[^ ^19^, ^20^ ^]^ Moreover, the (photocatalytic) activation of carbon dioxide on MoS_2_ was demonstrated, rendering these materials potential substitutes for noble metals in (photo‐)catalysis.^[^ ^6^, ^7^ ^]^ In addition, sulfur vacancies in MoS_2_ were suggested to be active as hydrogenation catalysts toward methanol.^[^ ^21^ ^]^ The structure and composition of supported and promoted MoS_2_ hydrotreating nanocatalysts along with potential activation and deactivation routes have been widely disentangled with the help of such well‐defined model systems and a combination of high‐resolution microscopy (scanning tunneling microscopy (STM)) with elemental spectroscopy (esp. X‐ray photoelectron spectroscopy (XPS)) from ultra‐high vacuum (UHV) to near‐ambient pressure conditions.^[^ ^22^ ^]^

In contrast to the other aforementioned MS_2_ compounds, Ti‐based materials specifically benefit from the advantage of a high natural abundance,^[^ ^23^ ^]^ high stability during reactions,^[^ ^24^ ^]^ and non‐toxicity.^[^ ^24^ ^]^ Surprisingly, titanium disulfide (TiS_2_) has not received a great deal of attention with respect to material or catalytic properties, despite its application as a dry lubricant and low‐density cathode material in lithium‐ion batteries (intercalation of Li^+^).^[^ ^25^, ^26^ ^]^ Nevertheless, there are a few investigations on single‐layer materials and systematic studies of the chemical (catalytic) properties of TiS_2_. For example, TiS_2_ monolayers have been investigated as ultrathin bifunctional catalysts for the hydrogen evolution reaction (HER) and oxygen evolution reaction (OER).^[^ ^27^ ^]^ Single‐atom‐doped TiS_2_ has been shown to be effective for the oxygen reduction reaction (ORR),^[^ ^28^ ^]^ and TiS_2_‐TiO_2_ heterostructures have been synthesized for enhancing photocatalytic activity.^[^ ^29^ ^]^ Recent electrochemical studies have demonstrated the conversion of CO_2_ into carbon monoxide with TiS_2_ films supported on a carbon paper electrode.^[^ ^8^ ^]^ Furthermore, TiS_2_ has been used as a catalyst for ammonia formation from N_2_, NO reduction, or furfural functionalization.^[^ ^30^, ^31^, ^32^ ^]^ In addition, the band gap of around 1.0 eV for bulk and 1.1 eV for monolayer TiS_2_ renders the potential of harvesting sunlight from the visible range of the solar spectrum.^[^ ^33^ ^]^ This semiconductive behavior is also the reason why TiS_2_ is utilized in some perovskite solar cells.^[^ ^34^ ^]^ Not only the photo‐ and electrocatalytic activity, but also corrosion and degradation mechanisms can be understood at the atomic level with the help of well‐defined model systems. This study presents the fabrication of such suitable models, which should foster future investigations regarding the physical and chemical properties of nanostructured TiS_2_.

Thus, one challenge is to establish a synthesis protocol for high‐quality, well‐defined TiS_2_ mono‐layer nanosheets on substrates that allow the application of state‐of‐the‐art microscopy and spectroscopy. Up to this point, mainly exfoliation from 1T bulk phases has been reported, which yields highly defective extended single‐ or multilayers.^[^ ^35^ ^]^ Only one previous study by Friend et al. reports the selective synthesis of few‐to‐ten nm‐sized single‐layer TiS_2_ triangular nanoparticles on an Au(111) surface and is therefore the single point of reference.^[^ ^36^ ^]^ These authors used one mbar of SO_2_ to fabricate an AuS surface layer and subsequently evaporated metallic Ti in UHV before annealing to improve crystallization. Besides, by using a more benign, non‐oxidizing S source that allows a synthesis recipe transfer to other substrates, we will illuminate distinct differences of the fabricated nanomaterials in comparison to that earlier study.

In this work, we present a synthetic approach to fabricate well‐defined, shape‐controlled TiS_2_ single‐layer nanosheets (in the 2H phase) on a Au(111) surface. Gold seems to be a suitable substrate to investigate sulfur‐containing model catalysts because of its relatively good symmetry match with many MS_2_ compounds, its ability to catalyze the decomposition of sulfur reagents, and the low reactivity with other compounds.^[^ ^37^ ^]^ In contrast to commonly used H_2_S or SO_2_, we use a benign liquid organic sulfur source (dimethyl disulfide (DMDS)), which is less toxic, thus being safer and easier to handle.^[^ ^38^ ^]^ Structural and compositional insights from atomic resolution scanning tunneling microscopy (STM) and synchrotron photoelectron spectroscopy (XPS) enable computational modelling to underline the importance of controlling the chemical potential of S to maintain shape control. As a side finding, monoatomic [Au]Ti_1_S_3_ nuclei are identified for the first time, likely as remainders from the sheet growth due to the occurrence of a minimum TiS_2_ size. These results not only add a nanostructured TMDC to the library of nanomaterials and essentially extend their understanding, but generally inform and guide future synthetic protocols, for example, by chemical vapor deposition. Finally, we herein build a platform to study the fundamental chemical properties of stochiometric and defective TiS_2_ down to the atomic level.

## 2 Results and Discussion

### 2.1 Overview: TiS_2_ Nanosheets on Au(111)

TiS_2_ nanosheets have been synthesized in ultrahigh vacuum (UHV) chambers by reactive deposition of titanium in a dimethyl disulfide (DMDS) atmosphere (≈ 1 ∙ 10^−7^ mbar) on the clean surface of an Au(111) single crystal, followed by annealing in the same DMDS atmosphere to improve the crystallization process. A mild thermal annealing in vacuum was applied to all samples to reduce the amount of residual S‐containing species on the bare gold surface. The nanosheets have been investigated with scanning tunneling microscopy and X‐ray photoelectron spectroscopy in UHV.

**Figure 1:**
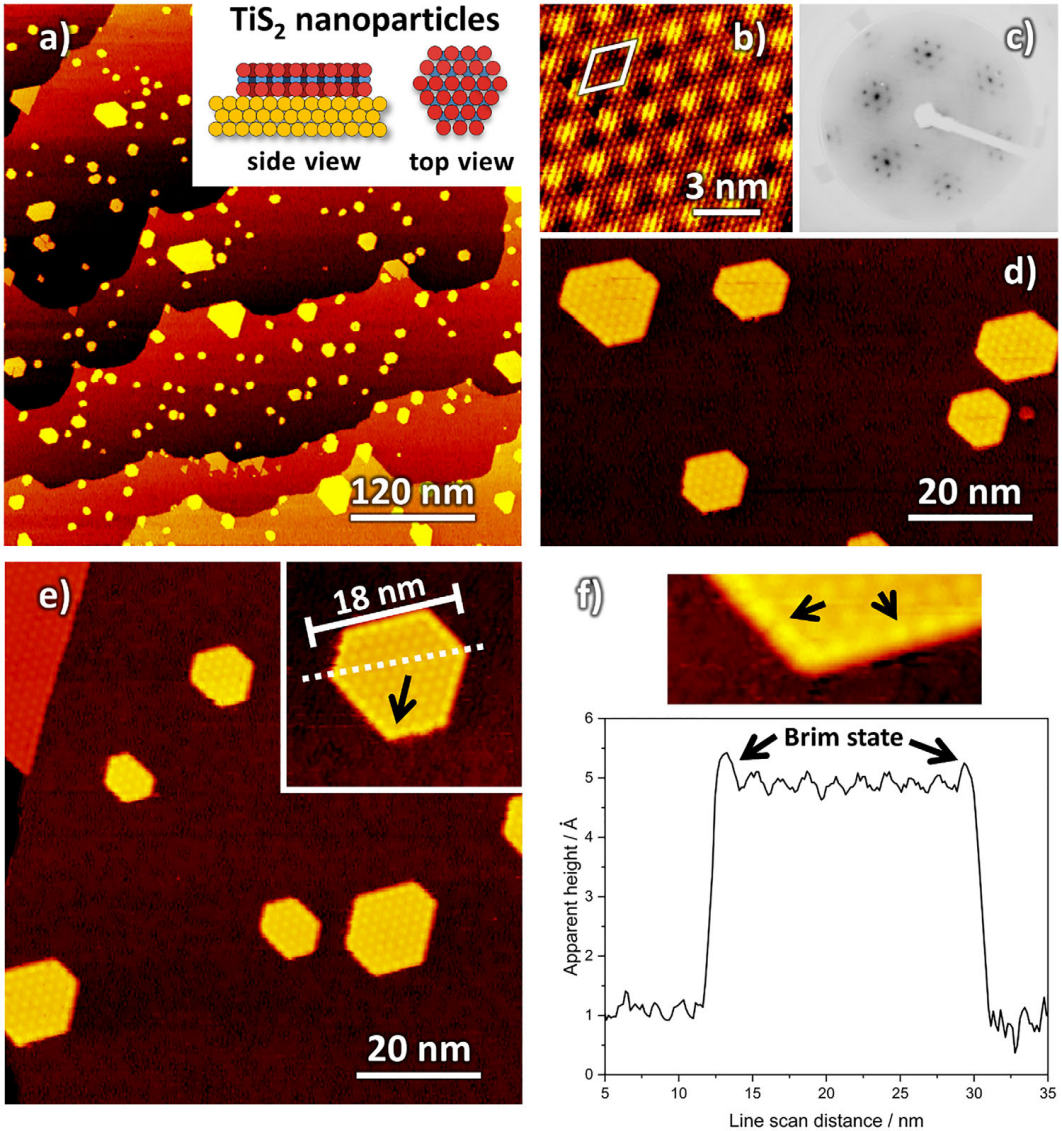
STM images showing single‐layer 2D TiS_2_ nanosheets grown on Au(111). a) The overview image (501 × 525 nm^2^) reveals a homogeneous distribution of the sheets on the gold terraces without any preferential growth position. The ball model was drawn to scale and is for illustration only (yellow = Au atoms, red = S atoms, blue = Ti atoms). Probing on top of the TiS_2_ nanosheets shows the characteristic 5‐on‐6 coincidence lattice in (b), which can also be observed in the LEED pattern in (c) (0.3 ML, 91.1 eV beam energy). d, e) (both 84 × 88 nm^2^) represent magnified views of hexagonally shaped TiS_2_ nanosheets. The edge of the sheets exhibits an increased contrast, indicating the appearance of a metallic edge, marked by black arrows (inset in (e) and in the corresponding line scan (f)).

First, a general overview of the fabricated TiS_2_ nanosheets will be given. **Figure** **1** shows a representative collection of STM images (0.1 ML TiS_2_ coverage, annealed at 615 K). The TiS_2_ nanosheets grow randomly distributed as single‐layer S–Ti–S sandwiches on the terraces and along the steps of the Au(111) substrate (Figure 1a). All sheets are grown in alignment with the gold surface.

The TiS_2_ nanosheets exhibit a characteristic moiré pattern (Figure 1b) widely independent of the sheet size and shape. The periodicity of the moiré pattern is 1.91 ± 0.03 nm, fitting to the 5‐on‐6 coincidence lattice, as also visible in the LEED pattern in Figure 1c (0.3 ML TiS_2_ coverage, 91.1 eV beam energy). The hexagonal unit cell exhibits a TiS_2_ (0001) lattice constant of 0.36 ± 0.01 nm. The lattice constants and LEED pattern are in good agreement with the earlier results of Friend et al.^[^ ^36^ ^]^ and are also comparable to bulk TiS_2_.^[^ ^39^ ^]^ It is noteworthy that no herringbone reconstruction on the bare Au(111) could be observed after any use of DMDS, indicating the presence of remaining S on the surface, lifting the herringbone reconstruction.^[^ ^40^ ^]^ The apparent height of the nanosheets is in a range between 1.4 and 3.8 Å, depending on the bias, but independent of the sheet shape (see Figure S2, Supporting Information).

Most of the sheets exhibit a hexagonal (22 %) or truncated triangular shape (43%) (for more details, see also Figure 4; Figure S1, Supporting Information). In contrast to an earlier report,^[^ ^36^ ^]^ just a minority of the sheets appear triangular (17%), and herein, the few observed triangles are exclusively located near the step edges of the Au(111) surface. Few other shapes, such as individual truncated sheets grown together or sheets without one of those equilibrium shapes, have been observed (18%). More than 85% of the sheets exhibit a size of up to 145 nm^2^, corresponding to a hexagonal sheet diameter of ≈5–15 nm (see histogram in Figure 4).

The edges of the TiS_2_ nanosheets show an increased STM contrast (≈+(0.1–0.4) Å in apparent height) at the outermost row of the moiré pattern (insets Figure 1e,f), indicating a metallic behavior of the edge. This metallic edge appears independent of the sheet shape and is comparable in apparent height, but also much broader than for MoS_2_, CoMoS, NiMoS, or WS_2_ sheets.^[^ ^2^, ^15^, ^16^ ^]^ Interestingly, this behavior was not observed by Friend et al.^[^ ^36^ ^]^ Such electronic properties of the metallic edge are often considered relevant as reactive sites in catalytic reactions, for example, for hydrogen dissociation on (promoted) MoS_2_, indicating a potential future field of application.^[^ ^41^ ^]^

**Figure 2:**
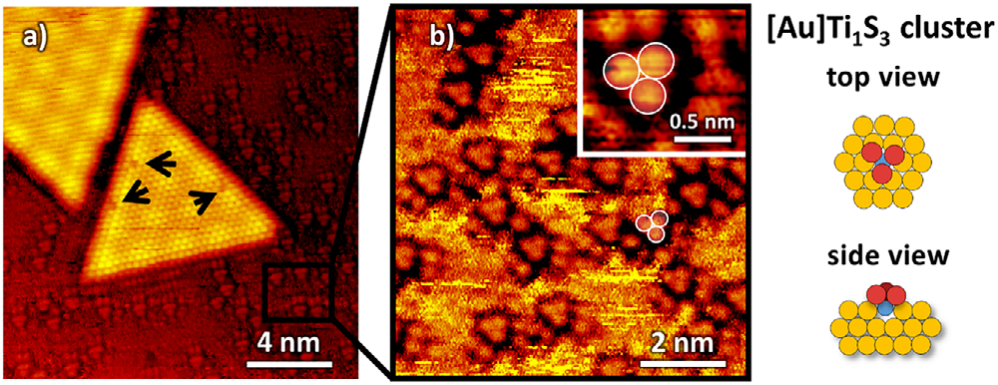
a) High‐resolution STM image of a TiS_2_ nanosheet shows single sulfur vacancies appearing on the edge and basal plane (17 × 18 nm^2^). b) STM image (8 × 9 nm^2^ (not the exact spot in (a)), inset 1.5 × 1.5 nm^2^) of [Au]Ti_1_S_3_ clusters on the Au(111) surface as shown in the ball model (yellow = Au atoms, red = S atoms, blue = Ti atoms).

Some of the TiS_2_ nanosheets also exhibit dark atomic‐sized features on the basal plane and edges, as shown in **Figure** **2**a (for the case of triangles, however, there was no preference for any shape). Because the S lattice is probed with STM, the location of the depression could indicate a missing S in the topmost layer of the TiS_2_ phase, a single sulfur vacancy. Such defects have also been recorded for bulk TiS_2_^[^ ^42^ ^]^ and other MS_2_ compounds, caused by exposing these compounds to reactive atmospheres or by artificial creation (ion bombardment), but for example not in the report of Friend et al.^[^ ^18^, ^36^, ^43^, ^44^ ^]^

A minimum side length for single‐layer TiS_2_ was identified, as the smallest observed nanosheets have a side length of nine S atoms, yielding three moiré hills. These sheets thus exhibit a triangular shape. In comparison, much smaller sheets with side lengths of, for example, only five S atoms have been observed for other sulfides such as MoS_2_.^[^ ^45^ ^]^ Thus, the moiré reconstruction appears to be of high importance for the stability of TiS_2_ on Au(111).

In addition to the new shapes, the defects and the critical side length of the TiS_2_ nanosheets, in contrast to the previous report of Friend et. al, small clusters have been found on the Au(111) surface next to the TiS_2_ nanosheets. These are randomly distributed on the surface as seen in the STM images in Figure 2a,b. The clusters consist of one single titanium atom with three S on top, denoted as [Au]Ti_1_S_3_ in the following. Based on reference experiments (see Figure S5, Supporting Information), in which first a dilute Ti in Au alloy^[^ ^46^, ^47^ ^]^ was created and subsequently exposed to DMDS at 500 K, the titanium atoms of these clusters are likely alloyed into the gold surface, similar to single‐atom alloys (SAAs).^[^ ^48^ ^]^ The bare gold surface often appears noisy between these well‐resolved clusters, which could indicate remaining mobile S species on the bare gold (Figure 2b). Mild annealing in UHV was used to reduce the amount of such species to improve STM imaging and reduce corresponding signals in S2p XPS spectra. However, such noise was never observed on the TiS_2_ sheets or over the [Au]Ti_1_S_3_ clusters_._

The presence of such clusters has not been described for other MS_2_ compounds on Au(111) except for TaS_2_.^[^ ^2^, ^5^, ^14^, ^15^, ^19^, ^37^ ^]^ In that case, the role of such nanoclusters was widely disentangled in an STM study by the group of Busse.^[^ ^37^ ^]^ There, single TaS_3_ nanoclusters embedded into the gold surface act as nuclei for an extended growth of sheets starting with monomers, but eventually begin to agglomerate to dimers, trimers, and larger structures.^[^ ^37^ ^]^ DFT calculations showed that TaS_3_ nanoclusters are more stable in contrast to TaS, TaS_2,_ or TaS_4_ clusters. However, herein, no stable agglomerates of such clusters ([Au](Ti_1_S_3_)_n_) were found. In combination with the finding of a minimum side length, it seems that forming a moiré pattern is essential for the stability of the TiS_2_ nanosheets. Thus, sheets with a size smaller than the critical side length of nine sulfur atoms probably cannot exist because they are unable to establish a stable moiré structure. As a consequence, [Au]Ti_1_S_3_ nucleates could be remainders from Ostwald ripening in which sheets with sizes below the critical side length of nine S atoms decompose back to nucleates.

**Figure 3:**
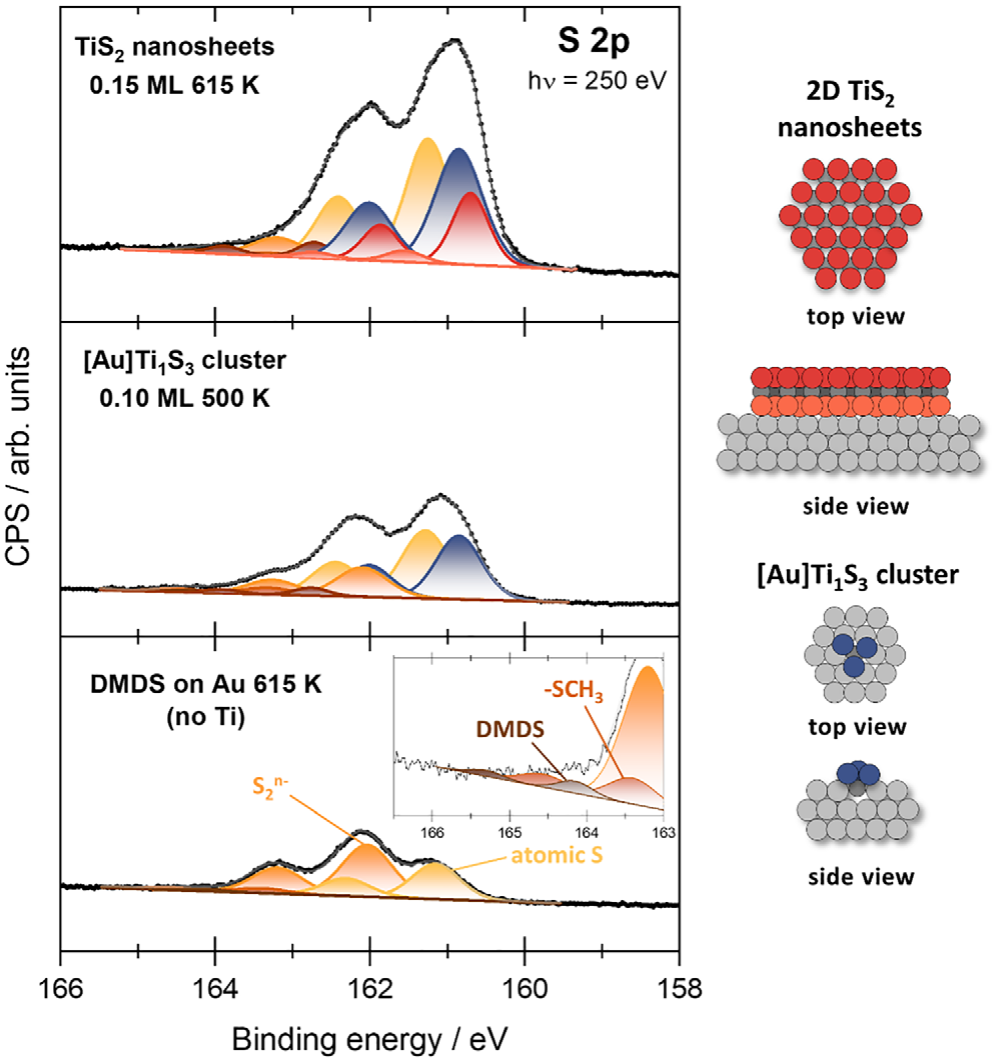
Deconvoluted XPS series starting with DMDS dosed on the clean Au(111) single crystal and annealing to 615 K (bottom + inset). The spectrum shows the decomposition of the DMDS to several sulfur species on the gold surface (assignment see Table 1). These species can also be observed after the synthesis of [Au]Ti_1_S_3_ clusters (middle). Here, one can clearly determine an additional component, yielding the signal position of the clusters. Finally, after the synthesis of TiS_2_ nanosheets (0.15 ML, 615 K), two additional signals appear (upper spectrum). The colored ball models on the right side represent the origin of the different components to differentiate between the S signals from TiS_2_ nanosheets and signals emerging from the sulfur of the [Au]Ti_1_S_3_ clusters.

To determine the compositional details of the TiS_2_ nanosheets, we acquired X‐ray photoelectron spectra at the MATLINE beamline at the ASTRID2 synchrotron (Aarhus, Denmark). The beamline allows for high resolution for rather soft photon energies (below ≈300 eV), while the resolution gets much lower at higher energies (up to ≈800 eV). For XPS recorded at a high photon energy (hν = 590 eV), the Ti 2p spectra show a broad, slightly asymmetric signal (Figure S4, Supporting Information, Peak at 456.3 eV, FWHM ≈ 3.0 eV, comparable to bulk TiS_2_ = 456.1–456.2 eV).^[^ ^39^, ^49^ ^]^ Due to the broad shape, this peak does not change enough within our experimental series to yield relevant information, but scales linearly with the deposited Ti coverage as expected. The S 2p core level (hν = 250 eV) provides more information (**Figure** **3**). In the S 2p region, each chemical species yields two signals due to spin‐orbit coupling (namely the S 2p_3/2_ and the S 2p_1/2_). The S 2p spectra have been deconvoluted with a spin‐orbit splitting of 1.16 eV, and constant peak positions and FWHM for each chemical species derived from reference experiments. More information on the XPS analysis and additional spectra can be found in the SI (S4). In the following, all positions will be given in binding energy for the more intense S 2p_3/2_ signal. One has to distinguish between three classes of signals, each consisting of one or more chemical species. A full list is given in **Table** **1**.

**Table 1:** Overview of the observed S 2p signals. All BE positions are given for the S 2p_3/2_ component.

	Species	Position / eV	Literature Position / eV
Decomposition of DMDS on Au	Atomic S	161.1–161.3	161.0–161.2^[^ ^50^, ^51^, ^52^ ^]^
	Disulfur complex S_2_^n‐^	162.0–162.2	162.3^[^ ^50^ ^]^
	Thiolate ‐SCH_3_	163.3–163.5	163.3–163.5^[^ ^50^, ^51^, ^52^ ^]^
	Readsorbed DMDS	164.2–164.5	164.4 for DMDS on Ni(111)^[^ ^52^ ^]^
	Unknown S species (only appears after flashing)	162.7–162.8	–
[Au]Ti_1_S_3_ cluster	[Au]Ti_1_S_3_	160.8‐160.9	–
TiS_2_ nanosheets	TiS_2_ upper basal plane	160.7	160.8 for bulk TiS_2_^[^ ^39^, ^49^ ^]^
	TiS_2_ bottom basal plane	161.5‐161.6	–

The bottom spectrum of Figure 3 represents the S 2p region after exposing DMDS to the bare gold surface (no Ti) and annealing to 615 K (analogous to the TiS_2_ synthesis, but without Ti). These signals originate from remaining S components on the gold surface and have been assigned based on literature reports and reference experiments. The DMDS decomposed under these conditions into several sulfur‐containing species. The two dominant species are atomic S (peaking at 161.1–161.3 eV) and a disulfur complex (S_2_^n‐^, 162.0–162.2 eV).^[^ ^50^, ^51^, ^52^ ^]^ Readsorption of DMDS from the chamber background leads to two minor signals, specifically molecularly adsorbed DMDS (peaking at 164.2–164.5 eV) and thiolate (from dissociation of the S─S bond upon adsorption, peaking at 163.3–163.5 eV). This assignment is based on UHV studies with other sulfur‐containing compounds on gold and DMDS on Ni(111).^[^ ^50^, ^51^, ^52^ ^]^ Flashing the sample after annealing to 615 K leads to a small additional peak appearing at 162.7–162.8 eV, which could not be assigned to a certain species. From the corresponding C 1s spectrum (see Figure S4, Supporting Information), we know that this species does not contain any carbon, which also applies to the species at 161.1 and 162.0 eV. Probably, the signal at 162.7 eV evolves from agglomerates of sulfur (stochiometry S_n_) as shown for other compounds.^[^ ^40^, ^53^ ^]^

The spectral contribution of [Au]Ti_1_S_3_ clusters was determined in another reference experiment by a selective synthesis to obtain only [Au]Ti_1_S_3_ clusters and no TiS_2_ nanosheets (description see Section S5, Supporting Information). In brief, first, a surface alloy of Ti in Au (0.1 ML) was formed, which was then exposed to DMDS at 500 K. Based on STM experiments (see Figures S5 and S6, Supporting Information), this yields only the small [Au]Ti_1_S_3_ clusters, but no extended TiS_2_ sheets. The corresponding spectrum displayed in Figure 3 (middle) shows an additional signal peaking at 160.8–160.9 eV, which is therefore assigned to those clusters. Unfortunately, there are no further reports about such nuclei to compare with literature values.

The upper spectrum in Figure 3 was recorded for the TiS_2_ nanosheets (615 K, 0.15 ML). The presence of the nanosheets results in two additional signals in the S 2p spectrum peaking at 160.7 and 161.5–161.6 eV. Similar to studies of MoS_2_ on gold,^[^ ^54^ ^]^ these species are assigned to different S locations on the nanosheets, which are the upper and the bottom basal plane, respectively, as indicated in the colored ball model in Figure 3. All signal assignments were confirmed by trends in the experimental series under variation of the TiS_2_ coverage, sheet size, and annealing temperature. The major signal at 160.7 eV is related to the S in the upper basal plane, which is in good agreement with earlier reports of bulk TiS_2_ (160.8 eV).^[^ ^39^, ^49^ ^]^ However, the signal of S from the bottom basal plane is shifted 0.8–0.9 eV toward higher binding energies due to the interaction with the gold surface. In addition, the intensity of that signal is much smaller than the signal from the upper basal plane (peak ratio upper:bottom ≈6:1) due to the limited mean free path of the photoelectrons (here, 250 eV photon energy yields ≈ 0.5 nm at 90 eV kinetic electron energy). A similar effect has been observed for MoS_2_ nanosheets on gold.^[^ ^55^ ^]^ Due to the comparably large size of the sheets, the spectra are dominated by the basal plane signals. The overall S:Ti ratios were ≈ 3.1–3.2:1 (determined by Al Kα lab source XPS and including all S species) in all coverage‐ and temperature‐dependent experiments (expected ratio between 2.5‐3:1 depending on [Au]Ti_1_S_3_ cluster concentration). This higher value can be explained by the readsorption of DMDS and other S species on the bare gold, leading to higher sulfur concentrations.

### 2.2 Size and Shape Control of TiS_2_ Nanosheets

**Figure 4:**
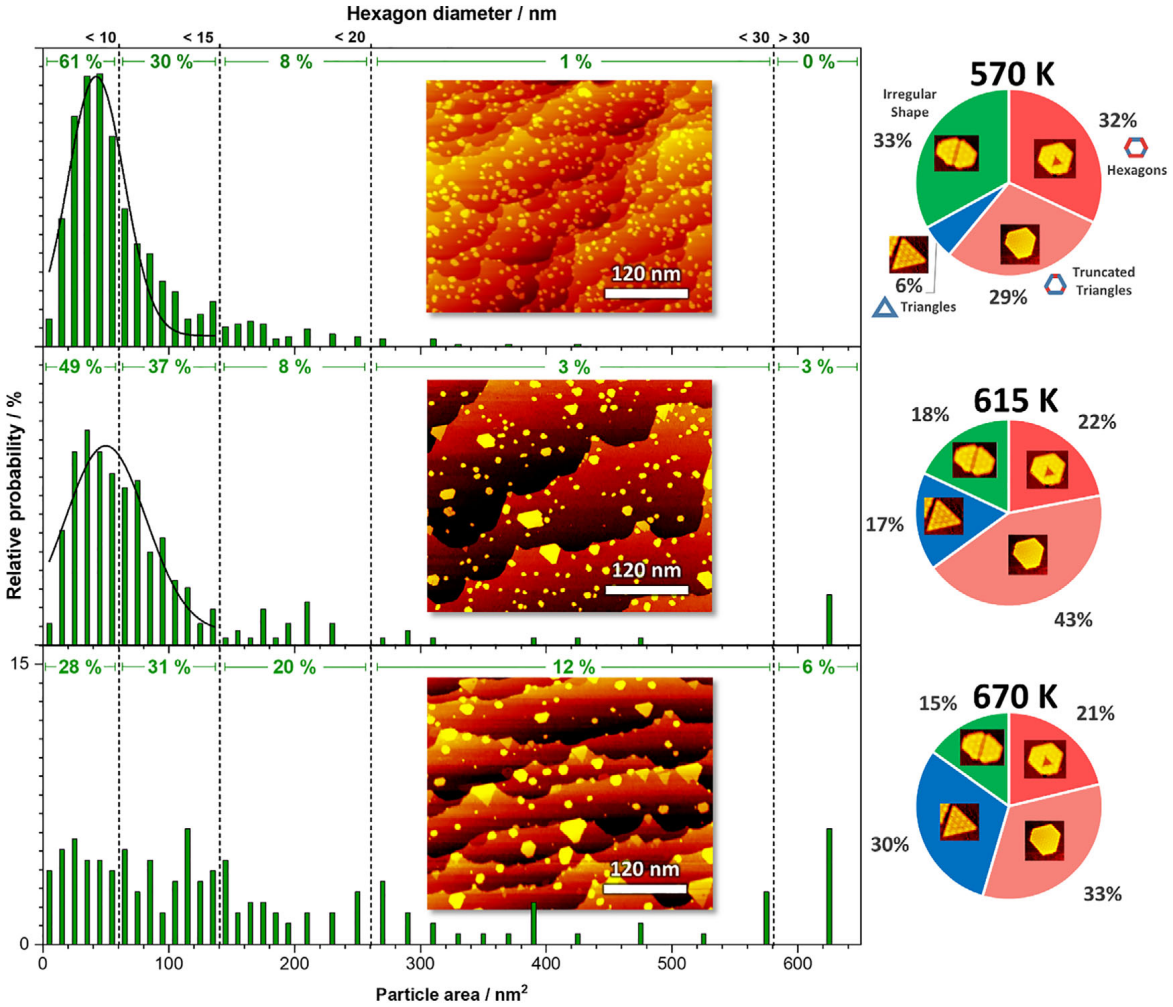
Area (histogram) and shape (pie chart) distribution of the TiS_2_ nanosheets after annealing in DMDS to 570, 615, and 670 K (0.1 ML each). The statistical analysis has been derived from the overview image and 15 further STM images (each 84 × 88 nm^2^), leading to 746 analyzed sheets at 570 K, 261 sheets for 615 K, and 177 sheets for 670 K. As a guide, the theoretical diameter for perfect hexagons is given at the top for the corresponding area.

In order to control the TiS_2_ nanosheet size and shape, we identified the annealing temperature and coverage as relevant parameters. First, the effect of the annealing temperature (after deposition at 300 K) was surveyed. Three different reaction temperatures (570, 615, and 670 K) were tested, inspired by common values for similar MS_2_ compounds.^[^ ^2^, ^5^, ^14^, ^15^, ^36^, ^37^ ^]^ Second, two higher coverages (0.21 and 0.33 ML) were tested while the annealing temperature was kept at 615 K. In order to determine trends in the sheet sizes and shapes, respectively, we present in **Figure** **4** an overview of the statistical analysis for different annealing temperatures based on area histograms and shape categorization. It can be seen that the sheet size depends strongly on the synthesis temperature, growing bigger with increasing annealing temperature as expected. Consequently, because of constant coverage, the number of sheets decreases with higher annealing temperatures. For a better overview, size categories were introduced in Figure 4. The most common size at 570 K is smaller than 65 nm^2^ (42 ± 22 nm^2^) with a share of 61 % corresponding to a theoretical diameter of hexagonal sheets of <10 nm. More than 90 % of the sheets are smaller than 145 nm^2^.

After annealing to 615 K, the growth of the sheets can be observed (48 ± 32 nm^2^). The number of sheets with a size between 65 and 145 nm^2^ (share 37 %) increased while the number of smaller sheets decreased (49 %). This trend continues for the annealing temperature of 670 K. Here, the most common area is between 65 and 145 nm^2^, with a share of 31 %. In addition, the share of sheets with a medium size (area between 145 and 585 nm^2^; 15 and 30 nm diameter) increases significantly from 11 % at 615 K to 32 % at 670 K. The share of large sheets with an area of 585 nm^2^ and more grows from 0 % at 570 K to 3 % at 615 K to 9 % at 670 K. The dependency of the TiS_2_ sheet size on the annealing temperature is evident. The higher the annealing temperature is, the larger the formed TiS_2_ nanosheets are. However, with increasing annealing temperature, the size distribution also broadens, which might be an effect due to limited growth kinetics of on‐surface ripening. Furthermore, the effect on sheet sizes is in agreement with the results of other MS_2_ compounds like MoS_2_, WS_2,_ and RuS_2_^[^ ^14^, ^15^, ^19^ ^]^ and the observations made by Friend et al.^[^ ^36^ ^]^ They observe a sheet size ranging from 3 to 30 nm at 670 K annealing temperature, whereas at 800 K, sheets with a size up to 80 nm have been reported.^[^ ^36^ ^]^ A similar behavior was observed for the coverage series (Figure S1, Supporting Information). Higher coverages lead to larger sheet areas, although more complex shapes occur due to the agglomeration of individually pinned sheets.

**Figure 5:**
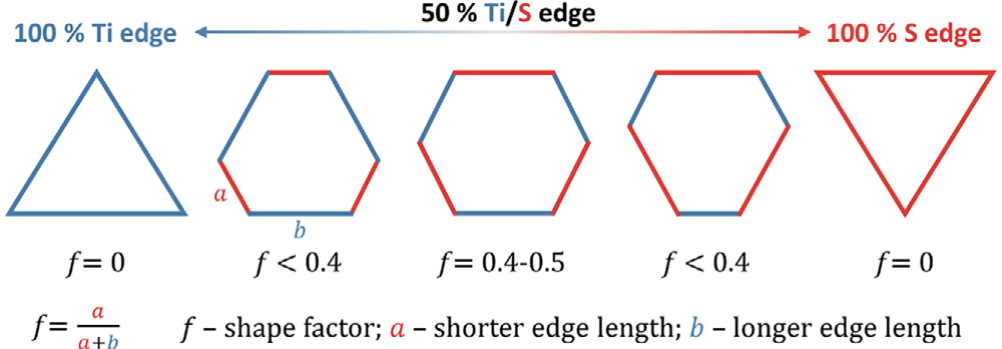
Categorization of nanosheets into different groups by the shape factor f. Blue lines represent Ti edge terminations (M‐edge), whereas red lines show the S edge termination (S‐edge). The sketch demonstrates that triangular sheets only reveal one edge termination (either Ti or S edge), whereas for truncated or hexagonal sheets, both edges are present.

One of the main remaining questions at this point is the following: Why do our TiS_2_ sheets appear in mostly hexagonal or truncated triangular shapes, in strong contrast to the earlier report by Friend et al., who found almost exclusively triangular shapes?^[^ ^36^ ^]^ As a first step, a quantitative shape analysis has been performed. For categorization, the side length of the sheets was measured manually, and based thereon the sheets were classified into four shape categories: hexagons, truncated triangles/nonsymmetric hexagons, triangles, and sheets with other shapes. The classification was performed based on a shape factor 𝑓, which was calculated from the measured side length assuming two types of edges (M‐edge and S‐edge) as described elsewhere.^[^ ^56^ ^]^ This nomenclature is commonly used for 2D transition metal sulfides in the 2H polymorph. While the S‐edge is terminated by lattice S sites, the M edge is constructed from lattice M (M = Mo, Ti, Ta, Co, …) sites, which are saturated by S dimers under sulfur‐rich conditions (fully sulfided). Under strongly reducing conditions, the S may be removed from this edge, exposing unsaturated M sites. By definition, a fully symmetric hexagon is defined with a shape factor of 0.5 (M‐edge and S‐edge have the same length) as shown in **Figure** **5**, while a triangle would exhibit a shape factor of 0 (only one type of edge). Herein, sheets with a shape factor of 0.4–0.5 were classified as hexagons. Truncated triangles or nonsymmetric hexagons were classified as one group due to their similar shape, with a shape factor 𝑓 < 0.4. Triangles were assigned manually (because 𝑓 =  0). In other words, symmetric hexagons have to be understood as a special case of truncated triangles. All other shapes were summed into the category of other shapes, in particular, merged sheets with defects, circular sheets or sheets without a defined shape. Note that the distribution of M and S edge lengths is directly connected to their edge formation energy by the Wulf construction.

The shape distribution of the sheets strongly depends on the annealing temperature, as evident from the pie charts in Figure 4. However, the step density during the experiments has not been considered during the shape analysis. The most common shapes for annealing at 570 K are sheets, with other shapes (33 %) in contrast to only 18 % at 615 K or 15 % at 670 K. The reason for this is probably the low annealing temperature, leading to non‐equilibrium shapes due to limited mobility on the gold surface and kinetic limitation of restructuring. Indeed, it was not possible to resolve a well‐defined crystallinity by STM for a minority of the sheets in the 570 K sample. A similar effect was observed for MoS_2_ nanosheets synthesized with DMDS and explained by an incomplete decomposition of DMDS (intact C─S bonds) and insufficient crystallization due to the low annealing temperature.^[^ ^38^ ^]^ However, we have not observed significant amounts of carbon on the 570 K sample, indicating that at least the C─S bonds are sufficiently dissociated herein. For higher coverages, more irregular shapes have been observed, likely due to the agglomeration of individual neighboring sheets during the growth.

In all samples herein, the sum of hexagonal and truncated triangles and nonsymmetric hexagons dominates the shape distribution (between 55 % and 65 %). The total share of hexagonal and truncated sheets (truncated triangles and nonsymmetric hexagons) reaches a maximum for 615 K with a share of 65 %. Based on our experiments, this is the optimal annealing temperature range to obtain hexagonally shaped TiS_2_ nanosheets, even at higher coverages (57 % for 0.21 ML and 59 % for 0.33 ML, see Figure S1, Supporting Information). For 670 K, the most common shapes are still the truncated (33 %) and hexagonal (21 %) shapes. A clear trend, on the other hand, was observed for the presence of triangular sheets, rising with the annealing temperature from 6 % to 30 % (570–670 K). A possible explanation could be that at higher annealing temperatures, one edge termination becomes more favorable than the other, which is necessary to form the triangular shape.^[^ ^36^ ^]^ However, it is intriguing that in contrast to the other shapes, the triangles were only observed at the steps of the gold substrate, while the gold terraces were predominantly covered by (nonsymmetric) hexagons and truncated triangles. Thus, step edges may play a role in the stabilization of triangular TiS_2_. This is also reflected in the fact that the share of triangular sheets shrinks with increasing coverage, due to increasing competition on the step edge positions (for 615 K: from 17 % at 0.10 ML and 13 % at 0.21 ML to only 3 % at 0.33 ML coverage).

The shapes observed herein are in contrast to the STM observations reported by Friend et al., who only observed triangular‐shaped TiS_2_ sheets, which they explained by a favored Ti edge termination.^[^ ^36^ ^]^ However, also based on that literature, the expected shape for TiS_2_ nanosheets is supposed to be a hexagonal (or truncated triangular) structure because of similar energies of Ti and S edge termination.^[^ ^36^ ^]^ Though it is challenging to differentiate between Ti and S edge termination in the STM, the observed hexagonal shapes indicate that in our case, coexistent Ti and S edge termination occur in roughly equal ratios at the TiS_2_ sheets. A similar hexagonal structure has only been observed for other MS_2_ compounds, such as MoS_2_ and WS_2_, under reducing conditions.^[^ ^14^, ^15^ ^]^ For example, exposing MoS_2_ nanoparticles to H_2_, a hydrogen‐induced reshaping into truncated triangles was observed.^[^ ^56^ ^]^ However, herein it seems that the reaction with DMDS as a sulfur source provides a chemical environment where both (Ti and S) edge terminations are energetically similar on the Au surface. We rule out a possible reduction from background H_2_, as the observed remaining S species on gold would be easily reduced by H_2_ at elevated temperatures. Nevertheless, the sheets are also stable after flashing the sample in UHV (short anneal to 610 K). No correlation between the shape of TiS_2_ on the terraces and defect sites (on the Au or on the nanosheets) was observed. The quality of the Au(111) surfaces before the synthesis of TiS_2_ was frequently checked with STM, and virtually no defects have been detected on the terraces.

**Figure 6:**
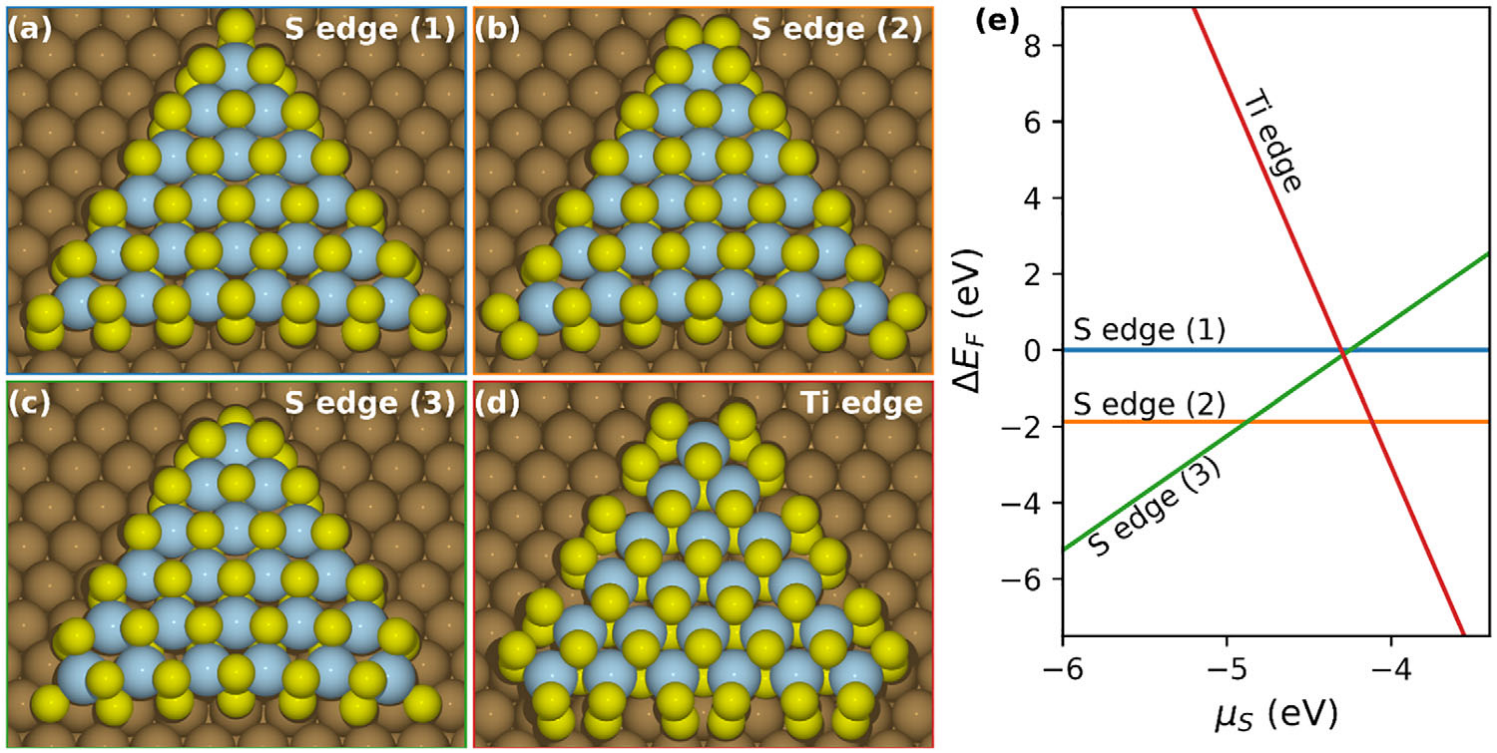
a–d) Four models of TiS_2_ nanosheets on Au(111) are considered in this work. Dark, yellow, and blue balls represent Au, S, and Ti atoms, respectively. e) Relative formation energy of TiS_2_ nanosheets on Au(111). Note that the outermost Ti edge in (d) is 100 % saturated with S atoms.

To obtain a deeper insight into the stability and formation energies of different edge terminations, which will ultimately govern the shape distribution, DFT calculations of the TiS_2_ nanosheets (2H structure) on Au(111) have been performed (see **Figure** **6**). We compared the relative formation energy of four TiS_2_ triangular‐shaped sheets on Au(111) and considered TiS_2_ nanosheets with different S edges as defined by their alignment with the gold surface (see Figure 6a–c) and Ti edge (see Figure 6d).

Since the S atoms at the corner of the S edge‐terminated sheets can be arranged in a few different ways, we constructed three models: 1) with S dimers at the corner aligned perpendicularly to the Au surface, 2) with S dimers at the corner aligned parallel to the Au surface, and 3) with S monomers. The models are shown in Figure 6a–c, respectively. The relative formation energies of the nanosheets as a function of the chemical potential of S (µ_*S*_) are shown in Figure 6e. We found that the preference of Ti edge or S edge depends strongly on the chemical potential of S. There is a region of µ_*S*_, at around −4.2 eV, where there is no preference for either S or Ti edge. This suggests that one can fine‐tune the chemical potential of S to obtain both terminations. While it is challenging to determine an accurate chemical potential during our experimental synthesis conditions, it may be roughly estimated from additional calculations of the relevant remaining S species on the gold surface (see Section S3, Supporting Information). As two examples, we considered S as a monomer and a dimer as two species present on the surface derived from the XPS assignment. The chemical potential of these species depends strongly on the annealing temperature (see Figure S3, Supporting Information), but it is in the range of a few negative eVs. The higher the annealing temperature rises and the lower the chemical potential gets, the more stable S edge terminations are over unstabilized Ti edges, leading to a preference for triangular‐shaped nanosheets with increased annealing temperature. These trends are in good agreement with our experimental findings of the annealing temperature series, in which a rising share of triangles was observed with consecutive increases of the annealing temperature, whereas the overall amount of hexagonally (or truncated triangularly) shaped sheets decreases. This strong dependence on the chemical potential of S as a descriptor for the TiS_2_ sheet shape also explains why Friend et al. obtained almost exclusively triangles, in contrast to this work. They used a surface layer of gold sulfide (from SO_2_ decomposition on Au(111)) as their sulfur source, which is expected to exhibit a very different chemical potential of S than a constant beam of an organic S donor. Thus, the results herein open up a tool for shape‐controlled synthesis approaches of early transition metal dichalcogenides.

## 3 Conclusion

In this work, we have presented a new synthetic protocol of 2D single‐layer TiS_2_ nanosheets on Au(111) using a mild, liquid organic sulfur source. The few nanometer‐sized sheets exhibit edges with an enhanced contrast in STM, similar to the metallic edges of (promoted) MoS_2_ and a few point defects, such as S vacancies on the edges and the basal plane. Additionally, [Au]Ti_1_S_3_ clusters coexist with the extended TiS_2_ sheets, which may act as remaining nuclei from the sheet growth.

The presence of multiple S‐containing surface species was demonstrated by synchrotron XPS. In addition to the four species on the bare gold surface (atomic and diatomic S, thiolates, and molecular DMDS), an S 2p signal position was determined for the [Au]Ti_1_S_3_ clusters. The lower basal plane of the TiS_2_ sheets yields S 2p signals that are shifted to higher binding energies in comparison to the upper basal plane sites, which appear similar to bulk TiS_2_, indicating a strong interaction with the gold substrate.

With the help of DFT modelling, we have shown that the chemical potential under synthesis conditions is a crucial descriptor for the shape of the nanosheets. Herein, hexagonal and truncated triangular sheets were dominating under all conditions, but in agreement with previous literature studies, a shift of the chemical potential by the annealing temperature can promote altered edge terminations, leading to the observed increase of triangular sheets at higher annealing temperatures.

## 4 Experimental Section

### Experimental Methods

The STM experiments were carried out in a custom‐built UHV system with two separated chambers, one used for the synthesis (base pressure 1 ∙ 10^−9^ mbar) and the other one (base pressure 1 ∙ 10^−11^ mbar) for imaging with a commercial Specs scanning tunneling microscope (Aarhus‐type NAP STM, used in UHV mode). The STM measurements were carried out with an etched W tip at different biases between ±50 mV and ±1 V applied to the sample and currents in the range of ±0.3–±3.0 nA at 300 K. No strong contrast changes depending on the scanning bias were observed. Clean Au(111) surfaces were prepared by two or more cycles of Ar^+^ ion sputtering (2 keV, 15 min) followed by annealing to 820 K in UHV for 20 –45 min. The surface cleaning was frequently checked by STM and/or XPS measurements. Ti was evaporated from rod material (purity 99.995 %) using a Mantis QUAD‐EVC electron beam evaporator (2 kV, 10.0 W, flux ≈ 0.7 nA). The evaporation rate was determined to be 0.03 ML min^−1^ by stepwise evaporation of Ti onto clean Au(111) in UHV at 300 K and subsequent coverage analysis via STM. For a typical TiS_2_ synthesis, the given coverage (typically 0.1–0.3 ML) of titanium was evaporated in a dimethyl disulfide (DMDS) atmosphere (≈ 1 ∙ 10^−7^ mbar) at 300 K. The DMDS (purity 99 %) was priorly cleaned by several conventional pump‐freeze‐thaw cycles. After the metal evaporation, the sample was heated to 615 K for 15 min while maintaining the DMDS atmosphere. Finally, the sample was flashed in UHV to 610 K for 3 min to remove DMDS and sulfur residues from the gold surface. The recorded STM images were edited by STM Image Analysis 2.4.4.0, starting with a rescaling of the images based on atomically resolved images of the clean Au(111) surface. Proceeding with a plane correction, a noise reduction by median filters has been used. The statistical analysis of the sheet shape was conducted by counting from 15 STM images (each 84 × 88 nm^2^), whereas for the size analysis, the overview images were additionally used. ImageJ was used to determine the number and size of the TiS_2_ nanosheets. The step density during the experiments has not been considered during the shape analysis. The LEED was recorded with an OCI Vacuum Microengineering LPS075‐D instrument (Beam energy 91.1 eV, in an analog UHV setup (FlexPES) at MAX IV Laboratory, Lund, Sweden).

The XPS spectra were recorded under UHV conditions at the MATLINE beamline of the ASTRID2 synchrotron at Aarhus University. The endstation (base pressure 3 ∙ 10^−9^ mbar) is equipped with a Phoibos 150 electron energy analyzer and an SX700 monochromator. We recorded the survey spectra of each experiment as well as core level spectra of the Au 4f, Ti 2p, S 2p, C 1s, and O 1s (parameters for each spectrum see SI) at room temperature. The precalibrated evaporation rate of Ti was confirmed by comparison to a combined STM and lab source XPS (1486 eV Al K‐α) coverage series. For this, the Ti/Au ratios acquired with 590 eV beam energy were corrected by the photoionization cross‐sections^[^ ^57^ ^]^ and the mean free path^[^ ^58^ ^]^ with respect to the Ti/Au ratios. As evident from the XP spectra, a minor readsorption of DMDS from the chamber background was noticed over time. For more details, see Supporting Information.

### DFT Calculations

Density functional theory‐based calculations were performed using the Vienna ab‐initio Simulation Package (VASP),^[^ ^59^ ^]^ employing the projector‐augmented wave (PAW)^[^ ^60^, ^61^ ^]^ and plane‐wave basis set. The generalized‐gradient approximation (GGA) was used in the form of the Perdew–Burke–Ernzerhof (PBE)^[^ ^62^ ^]^ functional, together with the DFT‐D3 correction^[^ ^63^ ^]^ to describe electronic exchange correlation. The electron kinetic energy cut‐off for plane‐wave expansion was set to 400 eV. Our model system consists of a triangular TiS_2_ cluster adsorbed on one side of a 4‐layered 10×10 Au(111) slab, which represents the Au(111) surface. We use a 15 Å vacuum added along the surface normal direction to decouple periodical images along this direction. Given the large size of the supercell (over 400 atoms), the Brillouin zone was sampled with one point at the zone center, using a Gaussian smearing with σ = 0.2 eV for structural relaxation. All electronic cycles converged to 10^−6^ eV. All structures were relaxed to minimize the stress until all components of forces acting on each atom were less than 10^−2^ eV Å^−1^.^[^ ^64^, ^65^ ^]^

The formation energy of a system is defined as:

(1) $$E_{F}=E-N_{Au}\mu_{Au}-N_{S}\mu_{S}-N_{Ti}\mu_{Ti}$$

where *E* is the total energy (calculated from DFT), *N_X_* and µ_*X*_ are the number of *X* atoms in the considered system and the chemical potential of *X* atom (*X =* Au, S, or Ti). As only the relative formation energy of the considered structures is of interest, instead of evaluating the absolute value of *E_F_*, one system was chosen as a reference and computed the relative formation energy Δ*E_F_* with respect to this system.^[^ ^66^, ^67^ ^]^ Since all considered systems had the same number of Au and Ti atoms, the Δ*E_F_* = Δ*E* − Δ*N_S_* µ_*S*_; where Δ*E* and Δ*N_S_* are the difference in the total energy (calculated from DFT) and the difference number of S atoms between the considered system and the reference system.

## Conflict of Interest

The authors declare no conflict of interest.

## Author Contributions

The manuscript was written through the contributions of all authors. All authors have given approval to the final version of the manuscript. N.K. did the investigation (lead), formal analysis (lead), and original draft preparation (lead). K.H., M.H., C.G. supported the investigation. D.L. did the theoretical investigation, software, formal analysis, and contributed to the original draft. T.S.R. did supervision, resources, review, editing and funding acquisition. J.V.L. did supervision, resources, review, editing and funding acquisition. L.M. lead supervision, conceptualization, review, editing, funding acquisition (lead).

## Supporting information



Supporting Information

## Data Availability

The data that support the findings of this study are available from the corresponding author upon reasonable request.
