# Supplementary material for: Monolayer TiS2 Nanosheets on Au(111)–Structural Characterization and Effect of Edge Stability for Shape Control
Source: Small. 2025 Aug 3;21(38):e06023. doi: 10.1002/smll.202506023 (PMC12462570; doi:10.1002/smll.202506023)
Supplement: Supplementary file 1 — Supporting Information [file SMLL-21-e06023-s001.pdf]

NANO · MICRO
small

Supporting Information

for *Small*, DOI 10.1002/smll.202506023

Monolayer TiS_2 Nanosheets on Au(111)–Structural Characterization and Effect of Edge Stability for Shape Control

*Niko Kruse, Kerry Hazeldine, Martin Hedevang, Celina Groothuis, Duy Le, Talat S. Rahman, Jeppe V. Lauritsen and Lars Mohrhusen**

Monolayer TiS_2 Nanosheets on Au(111) – Structural Characterization and Effect of Edge Stability for Shape Control

Supporting Information

Niko Kruse[1], Kerry Hazeldine[2‡], Martin Hedevang[2], Celina Groothuis[1], Duy Le[3], Talat S. Rahman[3], Jeppe V. Lauritsen[2], and Lars Mohrhusen[1]

[1]Institute of Chemistry, Carl von Ossietzky Universität Oldenburg, Carl-von-Ossietzky Straße 9-11, D-26129 Oldenburg, Germany

[2]Interdisciplinary Nanoscience Center (iNANO), Aarhus University, Gustav Wieds Vej 14, DK-8000 Aarhus, Denmark

[3]Department of Physics, University of Central Florida, 4111 Libra Drive, Orlando, FL 32816-2385, USA

[‡] Present address: imec, Kapeldreef 75, 3001 Leuven, Belgium

S1 Size and shape control of TiS_2 nanosheets depending on the coverage

Figure S1 shows the size and shape distribution for the different Ti coverages, all for annealing temperature of 615 K.

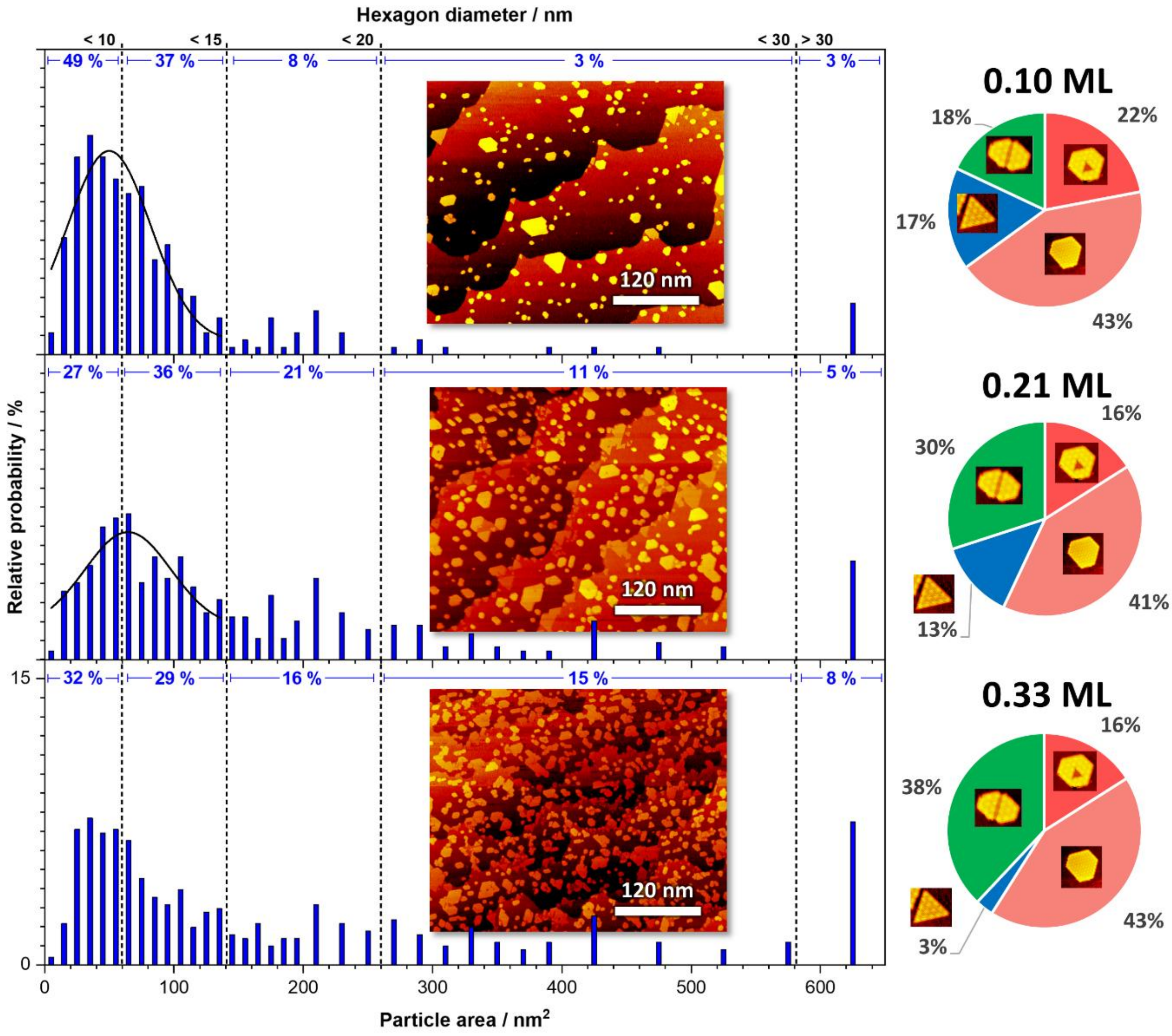

Figure S1: Size and shape (pie chart) distribution of the TiS_2 nanosheets after annealing in DMDS to 615 K at different coverages of 0.10 ML, 0.21 ML and 0.33 ML. Data for the statistical analysis has been derived from the overview image and 15 further STM images (each size 84 x 88 nm^2): 261 sheets at 0.10 ML, 445 sheets at 0.21 ML and 507 sheets at 0.33 ML.

In general, the samples with 0.21 ML and 0.33 ML exhibit relatively similar size distribution like the 0.10 ML experiment but reflect some trends: The share of small sheets up to 15 nm

(63 % for 0.21 ML and 61 % for 0.33 ML) is slightly lower than for the 0.10 ML coverage (86 %). Additionally, the share of medium-sized sheets (between 145-585 nm^2; 15-30 nm diameter) increases significantly with rising coverage. At 0.10 ML it is 11 %, rising with the coverage (0.21 ML is 32% and 0.33 ML is 31%). Finally, the share of large sheets is also more than twice as high as at 0.10 ML. This behavior is expected due to the merging of sheets at higher coverages. The share distributions of the 0.21 ML and 0.33 ML coverage are similar to shares of the 670 K annealing temperature for 0.1 ML, which reflects the fact of the formation of more extended sheets.

The total share of hexagonal and truncated nanosheets at both higher coverages (57 % for 0.21 ML and 59 % for 0.33 ML) does not change significantly compared to the share of 65 % at 0.10 ML. In addition, the share of triangular nanosheets shrinks from 17 % at 0.10 ML and 13 % at 0.21 ML to only 3 % at 0.33 ML coverage. On the other hand, the share of nanosheets with other shapes rises with increasing coverages from 18 % (0.10 ML) over 30 % (0.21 ML) to 38 % (0.33 ML). This is virtually expected due to the agglomeration of neighboring, growing (pinned) sheets with increasing coverage.

S2 STM investigations of apparent height

Bias-dependent measurements of the nanosheet height (see Figure S2) show that the apparent height of the sheets decreases at higher gap voltages. None of the obtained height values is large enough to support double-layer formation (>12 Å) or half-layer (S-Ti) stacks (<3 Å). As a further support for monolayer (S-Ti-S) sheets being in direct contact with the gold (111) surface, all sheets observed herein exhibit the same Moiré pattern, which is due (and therefore sensitive to) the stacking mismatch been substrate and 2D material on top.

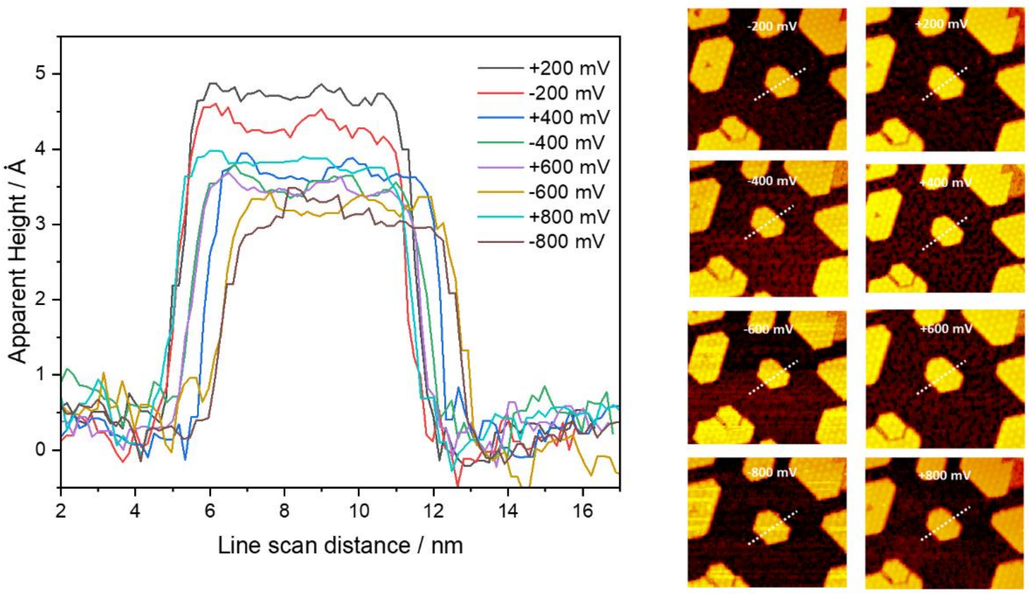

Figure S2: Bias-dependent height measurements of a TiS_2 nanosheet at constant current (I = 0.920 nA, 615 K, 0.33 ML).

S3 DFT studies: temperature dependency of chemical potential

It is challenging to determine the correct chemical potential under synthesis conditions in the experiment, especially since adsorption and dissociation of DMDS is involved. However, from DFT we could get a rough estimation based on the occurrence of remaining S species on the surface (as detected by XPS).

As we observed in the experiment that atomic S and dimers exist on the Au(111) surface, coexistent with the TiS_2 sheets, we assumed that the chemical potential of S in TiS_2 sheets is the same as that in S monomer or dimer. The chemical potential of S is then defined as:

$$\mu_S = \left[\left(E_{S/Au} - E_{Au}\right) + F_{S,Vib}\right]/n_S \qquad \text{Eq. S1}$$

Where $E_{S/Au}$ and E_{Au} are the total energy of system with n_S S atoms adsorbed on Au(111) and of bare Au(111). $F_{S,Vib}$ is the vibrational free energy contributed by the vibrational modes of S atoms.

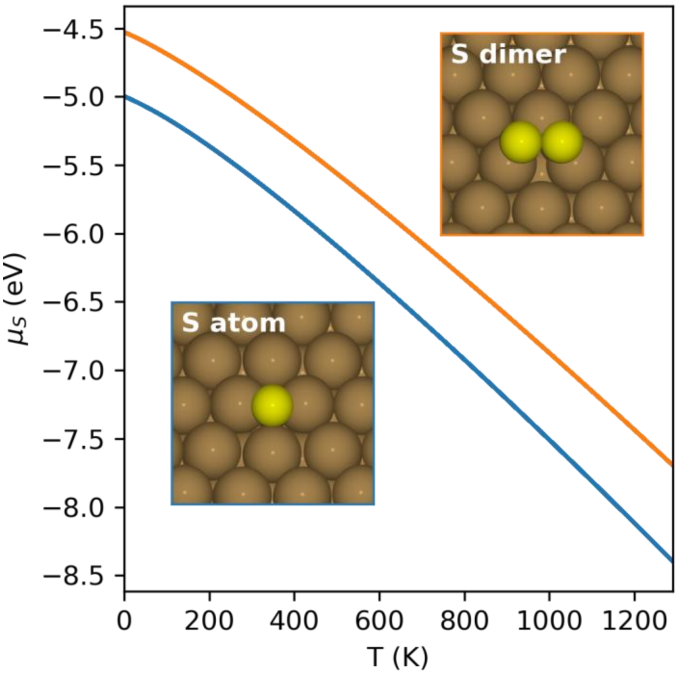

Figure S3: Chemical potential of S in monomer and dimer configurations depending on the annealing temperature.

S4 Further information and results of the XPS experiments

The XPS experiments were conducted at the MATLINE beamline of the Astrid2 synchrotron at Aarhus University. The endstation (base pressure $3 \cdot 10^{-9}$ mbar) is equipped with a Specs Phoibos 150 electron energy analyzer and a SX700 monochromator. Survey spectra were recorded with 590 eV excitation energy, while we used different energies to acquire surface sensitive core level spectra of the Au 4f, Ti 2p, S 2p, C 1s and O 1s. The acquisition details for each spectrum are given in Table 1:

Table 1: Acquisition parameter for the survey and core-level XP spectra.

Spectrum	Energy / eV	Scans	Step size / eV	Dwell time / s	Pass energy / eV
Survey	590	2	1	0.2	100
Ti 2p	590	10	0.05	0.2	20
O 1s	590	2	0.05	0.2	10
C 1s	375	3	0.05	0.2	10
Au 4f	250	2	0.05	0.1	10
S 2p	250	3	0.025	0.2	10

As we used the same Ti evaporator as for the STM experiments, the precalibrated evaporation rate of Ti was confirmed at low coverages by the Ti/Au ratios at 590 eV beam energy after correction for photoionization cross sections and mean free path compared to the Ti/Au ratios determined from a combined STM and lab source XPS (1486 eV Al Kα) coverage series. The recorded spectra were deconvoluted in Casa XPS using Gaussian/Lorentzian-shaped functions (70:30) except for the Au 4f signal (Gaussian/Lorentzian 90:10 with tailing (T = 2)). We used a spin-orbit splitting of 1.16 eV for S 2p and 3.70 eV for Au 4f. The peak position and FWHM for each chemical species were kept constant. A representative survey and a collection of core-level spectra (615 K, 0.1 ML sample) are presented in Figure S4.

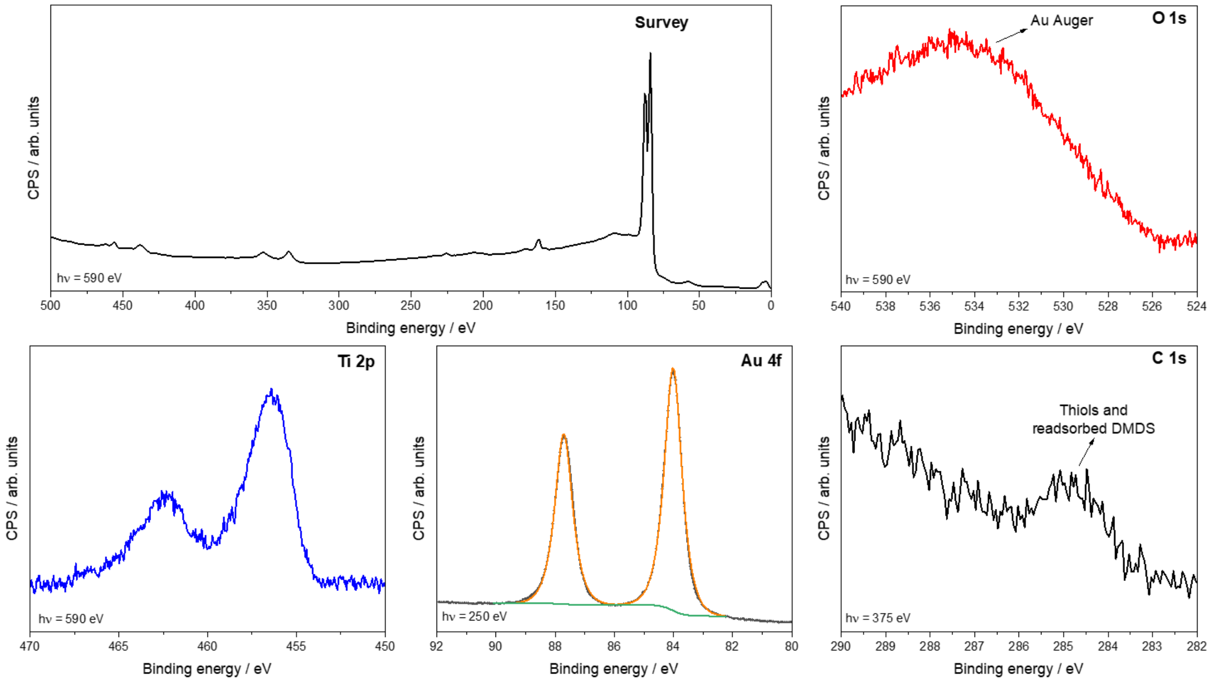

Figure S4: Survey spectrum and core level spectra of oxygen (O 1s), titanium (Ti 2p), gold (Au 4f) and carbon (C 1s). Note, that while no O is present on the sample surface, slow readsorption of DMDS from the chamber background leads to the observed signal in the C1s region (confirmed by the correlation of S2p and C1s spectra at different temperatures).

The survey spectrum shows the expected peaks related to the gold substrate and the TiS_2 nanosheets. The strongest signal complex at 84 eV is related to the Au 4f. Additional gold signals appear at 57 eV (Au $5p_{3/2}$), 110 eV (Au 5s), 335 eV (Au $4d_{5/2}$) and 353 eV (Au $4d_{3/2}$) in the survey. The Au 4f core level spectrum shows just a single chemical species peaking at the expected position of 84.0 eV. In the region around 455 eV, we observed a broad, slightly asymmetric Ti 2p signal (position = 456.3 eV, FWHM = 3.0 eV, comparable to bulk TiS_2 = 456.1-456.2 eV[1,2]). Deconvolution of this signal is challenging and leads to large error margins due to the large peak width, which is due to the poor performance of the synchrotron light source at these higher photon energies.

In addition, the Ti 2p signal did not change significantly during the experiments making it tough to gain relevant information about the [Au]Ti_1S_3 clusters and TiS_2 nanosheets. In contrast, the

S 2p spectrum provided more information, so we focused on the S 2p region in the main text. Based on conventional Al Kα lab-based XPS and the synchrotron-level XPS herein, our TiS_2 sheets are free of carbon. The C 1s region just shows a small signal, which is not scaling with the amount of titanium. Based on the correlation to the S 2p signal of reabsorbed DMDS and/or thiolates as well as the disappearance at elevated temperatures, this C 1s is assigned to reabsorbed DMDS from the chamber background. During all experiments, we were not able to detect any oxygen in our sample. The broad signal in the O 1s range originates from an Au Auger signal.

S5 Investigations of [Au]Ti_1S_3 clusters

Additional experiments were performed to clarify the structure of the observed clusters on the gold surface. Based on their geometric appearance, these clusters are [Au]Ti_1S_3, which could be nuclei for TiS_2 nanosheets. Similar TaS_3 clusters on the gold surface were studied by the group of Busse.[3] Therein, single TaS_3 nanoclusters act as nuclei for an extended growth of sheets by continuous agglomeration. Herein, an experimental sequence similar to that of the Busse group revealed that the titanium of these clusters is very likely embedded into the gold surface.[3] First, the alloying behavior of titanium was investigated by STM and XPS starting with the evaporation of titanium (metal) on the clean Au(111) surface (see Figure S5a) at 300 K (see Figure S5b) followed by annealing to 615 K, in the absence of any sulfur donor (see Figure S5c).[3] This acts as a mockup of the TiS_2 synthesis but without an S donor.

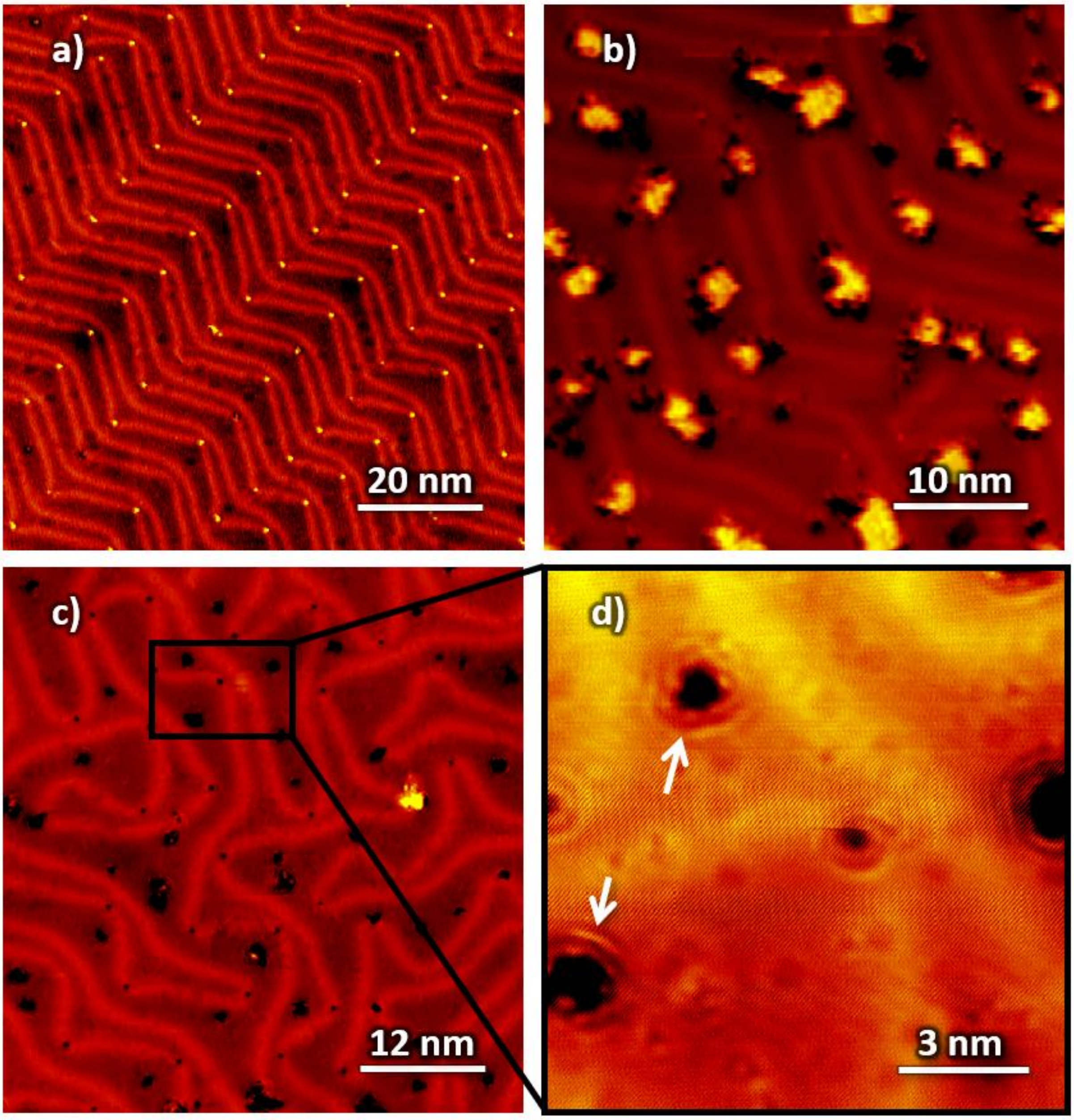

Figure S5. STM images of Ti alloying on Au(111) and $[Au]Ti_1S_x$ cluster formation. (a) shows a clean Au(111) surface (84 x 88 nm^2) with the characteristic herringbone reconstruction. (b) (37 x 42 nm^2) after titanium evaporation (0.1 ML at 300 K), Ti islands are visible as protrusions appearing in the STM. (c) represents the sample after annealing of the Ti islands in (b) to 615 K (50 x 53 nm^2) in the absence of any S donor. The herringbone pattern is affected by the titanium, now twisting around the titanium (or Au-Ti alloy) patches (dark features). High-resolution images of the alloyed titanium spots after

annealing such as in (d) (12 x 12 nm^2) reveal an interesting electronic environment similar to Friedel oscillations, marked by the arrows.

Figure S5a shows the clean Au(111) surface with the characteristic herringbone reconstruction. As described elsewhere, the elbows of the herringbone pattern often act as nucleation sites due to their locally increased electron density.[4,5] Indeed, the formation of titanium nanoparticles on the elbow sites can be observed after the deposition of 0.1 ML Ti at room temperature without annealing (inset in Figure S5b). These particles appear as protrusions with higher apparent height and do not significantly impact the herringbone reconstruction. However, around the particles, there is a zone of lower contrast, which might indicate the formation of an alloy or a zone of lower electron density at the titanium-gold interface and perimeter sites.

To investigate the alloying into the gold substrate, such "Ti nanoparticles at the gold surface" were subsequently annealed at 615 K (typical TiS_2 synthesis temperature) in UHV yielding the STM image in Figure S5c. Now, a displacement of the herringbone pattern can be observed. The new corrugation is twisted around some low-contrast features with a size between 0.5 nm and 2 nm on the surface, which is probably the evaporated titanium or a Ti-Au alloy now being embedded into the surface layer. Similar depressions in STM images were obtained by probing an alloy of gold atoms on Ni(111).[6] A closer view of these features is given in Figure S5d. An interesting electronic environment was observed around these patches, exemplarily marked by arrows beside the dark features on the gold surface (see Figure S5d). These features are likely caused by standing electron waves in occupied surface states, commonly called Friedel oscillations.[7,8] However, comparing samples before and after annealing, the titanium patches seem to be embedded into an Au(111) surface after annealing in contrast to the nonannealed sample, resulting in a lower tunneling current due to a decreased local density of states (LDOS), where Ti is present.[9] The alloying of titanium was also described in the literature previously,

showing similar depressions on the Au(111) surface due to embedded titanium in the STM images and line scans.[10,11]

Finally, to investigate the formation of Ti_1S_x nanoclusters, DMDS (at 500 K, 15 minutes, $1 \cdot 10^{-7}$ mbar) was dosed onto the alloyed surface obtaining the STM image in Figure S6a. Due to the low temperature, the formation of extended TiS_2 sheets can be prevented.

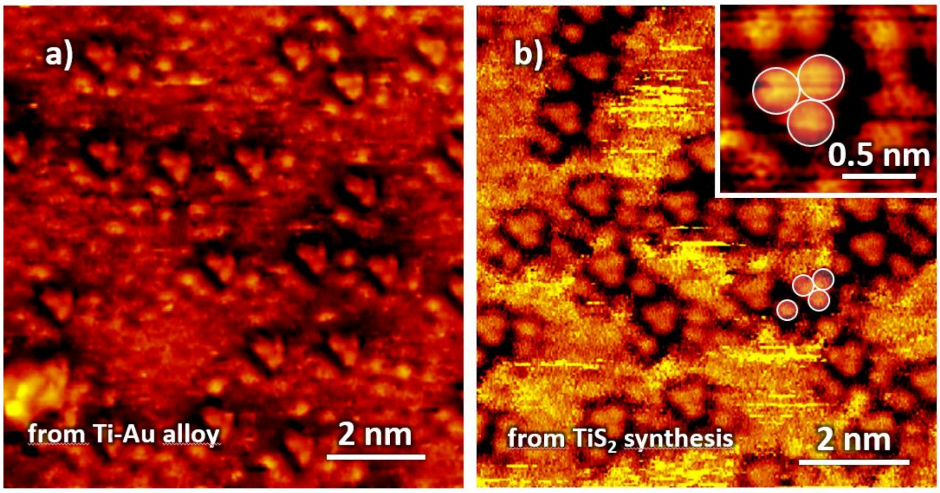

Figure S6: Synthesized [Au]Ti_1S_3 after dosing DMDS on alloyed Ti on Au(111) (a) in comparison to the nanoclusters observed during TiS_2 nanosheets synthesis (b) (both 8 x 9 nm^2).

Here, small single and three-atomic-appearing nanoclusters have been observed, fully resembling the previously observed nanoclusters obtained in the TiS_2 synthesis (see Figure S6b) in size, structure and apparent height. Interestingly, the concentration of clusters does not depend on the annealing time and, for TiS_2 sheets, the annealing temperature, which supports the assumption of a critical size for the TiS_2 nanosheet formation.

REFERENCES

[1] H. Martinez, C. Auriel, M. Loudet, G. Pfister-Guillouzo, *Appl. Surf. Sci.* **1996**, *93*, 231.

[2] K. Chen, M. Song, Y.-Y. Sun, H. Xu, D.-C. Qi, Z. Su, X. Gao, Q. Xu, J. Hu, J. Zhu, R. Zhang, J. Wang, L. Zhang, L. Cao, Y. Han, Y. Xiong, *Appl. Phys. Lett.* **2020**, *116*, 121901.

[3] T. Chagas, K. Mehlich, A. Samad, C. Grover, D. Dombrowski, J. Cai, U. Schwingenschlögl, C. Busse, *J. Phys. Chem. C* **2023**, *127*, 5622.

[4] S. Helveg, J. V. Lauritsen, E. Laegsgaard, I. Stensgaard, J. K. Norskov, B. S. Clausen, H. Topsoe, F. Besenbacher, *Phys. Rev. Lett.* **2000**, *84*, 951.

[5] J. Lauritsen, S. Helveg, E. Lægsgaard, I. Stensgaard, B. Clausen, H. Topsøe, F. Besenbacher, *J. Catal.* **2001**, *197*, 1.

[6] F. Besenbacher, J. V. Lauritsen, S. Wendt, *Nano Today* **2007**, *2*, 30.

[7] J. Fransson, A. V. Balatsky, *Phys. Rev. B* **2007**, *75*, 195337.

[8] K. Kanisawa, M. J. Butcher, H. Yamaguchi, Y. Hirayama, *Phys. Rev. Lett.* **2001**, *86*, 3384.

[9] A. Vojvodic, J. K. Nørskov, F. Abild-Pedersen, *Top. Catal.* **2014**, *57*, 25.

[10] J. Biener, E. Farfan-Arribas, M. Biener, C. M. Friend, R. J. Madix, *J. Chem. Phys.* **2005**, *123*, 94705.

[11] D. V. Potapenko, R. M. Osgood, *Nano Lett.* **2009**, *9*, 2378.
